# BRAF inhibitors in BRAF V600E-mutated ameloblastoma: systematic review of rare cases in the literature

**DOI:** 10.1007/s12032-023-01993-z

**Published:** 2023-04-28

**Authors:** Marcel Ebeling, Mario Scheurer, Andreas Sakkas, Sebastian Pietzka, Alexander Schramm, Frank Wilde

**Affiliations:** 1 Department of Oral and Plastic Maxillofacial Surgery, Military Hospital Ulm, Academic Hospital of the University of Ulm, Oberer Eselsberg 40, 89081 Ulm, Germany; 2 Department of Oral and Plastic Maxillofacial Surgery, University Hospital Ulm, Albert-Einstein-Allee 10, 89081 Ulm, Germany

**Keywords:** Ameloblastoma, BRAF, Facial reconstruction, Chemotherapy, Dabrafenib, Vemurafenib

## Abstract

**Background:**

Ameloblastoma in 66% of the cases harbor a somatic mutation of the “mitogen-activated protein kinase” signaling pathway (BRAF V600E). In V600E mutations, BRAF is in the permanent “on” state and relays the growth-promoting signals independently of the EGFR pathway. Therefore, mutant BRAF represents a target for handful of new drugs.

**Methods:**

We conducted a literature search, with the search terms “Vemurafenib, Dabrafenib, Ameloblastoma, and BRAF.” These included seven case reports with nine patients who underwent monotherapy with Dabrafenib or Vemurafenib or combination therapy with Dabrafenib and Trametinib.

**Results:**

The patients age ranges from 10 years up to 86 years. The distribution of women and men is 4:5. Patients with an initial diagnosis of ameloblastoma, as well as recurrences or metastasized ameloblastoma were treated. Indications cover neoadjuvant therapy up to the use in metastasized patients in an irresectable state. Results ranging from “only” tumor size reduction to restitutio ad integrum.

**Conclusion:**

We see the use of BRAF Inhibitors to reduce tumor size with consecutive surgical treatment as a reasonable option for therapy. However, we are aware that at present the data are based only on case reports with the longest follow-up of just 38 months. We encourage further clinical trials in the use of BRAF Inhibitors for selecting ameloblastoma patients in a multi-center setting.

## Introduction

In patients with newly diagnosed cystic lesions of the jaws, the practitioner must always consider an ameloblastoma as a potential diagnosis.

Ameloblastoma show a relatively high recurrence rate, especially in cases of resection without sufficient safety margins [1].

The gold-standard therapy for ameloblastoma is still radical surgical resection with sufficient safety margins between 1.5 and 2 cm and simultaneous reconstruction [1–7]

During the histological examination, a molecular biological examination and DNA sequencing of the tissue sample can be performed. Despite the limited data available to date, a somatic mutation of the “mitogen-activated protein kinase” signaling pathway has been detected. In particular, the BRAF V600E mutation, which is a single-nucleotide mutation with substitution of glutamic acid for valine and is apparent in about 2/3 of the cases [8–12], leads to the activation of the downstream MEK/ERK pathway, evasion of senescence and apoptosis, tissue invasion and metastasis, as well as the evasion of immune response [13].

The BRAF gene encodes the BRAF protein, a serine/threonine protein kinase [14]. BRAF is an essential link in the EGFR (Epidermal Growth Factor Receptor) signal transduction pathway. The activated enzyme-coupled EGF receptor (type I receptor) can bind several enzymes at their SH regions. One of them is the adapter protein Grb2—it binds the GEF exchanger SOS via an SH3 domain. The Grb2-Sos complex now formed is used by the receptor as an “extended arm” to convert the inactive Ras-GDP into the active Ras-GTP [15]. Ras-GTP is then able to recruit and activate the cytosolic serine/threonine kinase Raf at the cell membrane. The Raf protein then activates the kinase MEK (MAP/Erk kinase), which in turn phosphorylates tyrosine and threonine residues. MEK phosphorylates and thus now activates MAP kinase. The activated MAP kinase in turn phosphorylates or activates cytosolic and nuclear enzymes. It translocates to the nucleus and phosphorylates further downstream transcription factors, which then drive the cell cycle and cell differentiation. After deactivation, they redistribute in the cytosol and are available for further activation cycles [16].

An important substrate of MAP kinase is the transcription factor Elk-1, which forms a complex with SRF (Serum Responsive Factor). It binds to an SRE-type regulatory DNA sequence and activates the transcription machinery. Thus, additional transcription factors such as c-Fos and c-Jun are transcribed, which then initiate a second wave of transcription as the Fos-Jun complex and stimulate the cell to differentiate and proliferate [17].

Thus, the activated EGFR transduction pathway forces proliferation and prevents apoptosis [18]. In this signaling pathway, BRAF functions as a regulated molecular switch that is switched to the “on” state. In many tumors (e.g., colorectal carcinomas, malignant melanomas, thyroid carcinomas, NSCLC, and others), BRAF is in the permanent “on” state due to an activating V600E mutation in the BRAF gene and relays the growth-promoting signals independently of EGFR pathway activation—the signaling cascade is uncoupled from EGFR by mutant BRAF. Thus, blocking EGFR with anti-EGFR therapies in tumors with an activating BRAF mutation fails to silence EGFR signaling [19, 20]. On the other hand, mutant BRAF represents a target for handful of new drugs.

These include, for example, Dabrafenib or Vemurafenib, as selective, reversible inhibitors of RAF kinases. Both drugs are approved by the European Medicines Agency for the treatment of metastatic and non-resectable malignant melanoma, respectively. Dabrafenib has additional approval for the therapy of advanced non-small cell lung cancer in combination with Trametinib. The most common side effects of both drugs (which may affect more than 3 in 10 patients) are arthralgias, fatigue, rash, photosensitivity reactions, nausea and vomiting, alopecia, diarrhea, headache, pruritus, skin papillomas, and hyperkeratosis. The most common serious side effects include the development of squamous cell carcinoma, keratoacanthoma, and increase in liver enzymes [21, 22].

Since both drugs are currently not approved for the treatment of BRAF-V600E-mutated ameloblastoma, only isolated case reports on off-label use can be found in the literature. Therefore, we would like to summarize the described cases, present the effects and results, and give a hopeful outlook into the future of drug or at least combined drug-surgical therapy of ameloblastoma in this paper.

## Review of the literature

The literature search we conducted in PubMed, Ovid, Web of Science, Science direct, and Scopus yielded a total of just 7 papers with the search terms “Vemurafenib, Dabrafenib, Ameloblastoma, BRAF.” These include seven case reports with nine patients who underwent monotherapy with Dabrafenib or Vemurafenib or combination therapy with Dabrafenib and Trametinib (Table 1).

**Table 1:** Overview of the case reports found in the literature

References	Clinical setting	Tumor staging	Localization	Treatment	Duration of treatment	Side-effects	Outcome
Brunet et al.	26-year-old female	Recurrent and metastatic	Right mandible, tooth 48	Dabrafenib 150 mg (2×/day) + Trametinib 2 mg (1×/day)	30 weeks	None reported	Competed response after 12 weeks/complete remission after 30 weeks
Tan et al	85-year-old male	Recurrent, locally advanced	Left mandible, teeth 18 and 19	Dabrafenib 150 mg (2×/day) + left mandible composite resection with titanium plate placement and pectoralis major skin paddle	73 days	Plaque-like skin lesions on the back and scalp/thickening voice	After 16 weeks dramatic response with over 90% tumor volumen reduction
Frederic et al.	40-year-old male	Recurrent and metastatic	Left mandible	Dabrafenib 150 mg (2×/day) + Trametinib 2 mg (1×/day)	20 months	None reported	After 8 weeks complete disappearance of fluorodeoxyglucose activity in the lungs and reduction of the tumor mass in the face, jaw, and neck
Faden et al.	83-year-old female	Locally advanced	Right mandible	Dabrafenib 75 mg (2×/day), reduction by 50% given the patient’s comorbidities and overall functional status	12 months	None reported	After 8 months a safe swallow of all consistencies was possible/MRI demonstrated a 75% reduction in tumor volume
Hirschhorn et al.	15-year-old male	Locally advanced	Right mandibular ramus and body	Dabrafenib 4.5 mg/kg/day divided bid 150 + 75 mg/complete tumor resection	20 months	Fever/abnormal hair texture	No evidence of disease after 38-month follow up, mandibular contour near pre-disease state
	13-year-old male	Locally advanced	Right mandibular ramus and body	Dabrafenib 4.5 mg/kg/day 100 mg bid/complete tumor resection	16 months	Fever/acneiform rash	No evidence of disease after 38-month follow up, mandibular contour near pre-disease state
	10-year-old male	Locally advanced	Left mandibular ramus and body	Dabrafenib 4.5 mg/kg/day 100 mg bid/complete tumor resection	15 months	Erythema nodosum/folliculitis	No evidence of disease after 38-month follow up, mandibular contour near pre-disease state
Morgane Broudic-Guibert et al.	33-year-old female	Recurrent and metastatic	Left mandible	Vemurafenib 960 mg (2×/day), followed by reduction to 720 mg (2×/day) and second reduction 480 mg (2×/day)	None reported	None reported	After 3.5 months, a 30% decrease in the sum of the diameter of the lung target lesions/improvement in respiratory function with a decreased dyspnea
Gustavo S. Fernandes et al.	29-year-old female	Locally advanced	Ascending branch of the left mandible	Vemurafenib 960 mg (2×/day)	None	Grade one anorexia/nausea/fatigue	MRI shows a reduction of the lesion size to 18 × 13 × 14 mm

Remarkable facts	Symptoms/clinical findings	Radiological findings	Age of onset	First diagnosis	Initial therapy
None	Hemoptysis/cough	Numerous bilateral nodules	13 years	No	Surgery with clear margins
BRAF-V600E expression was lower in the treated specimen than in the untreated specimen/substantially greater areas of squamous differentiation in the treated specimen/the bulk of the response within the soft tissue component was more centrally located, leaving a rim of variable tumor surrounding a cystic space/tumor within bone appeared to be less responsive	4.5-cm tumor in the left angle of the mandible/pathologic fracture	40-mm left mandibular osteolytic lesion, containing enhancing soft tissue in the internal components	85 years	No	Enucleation with bone grafting
None	Tumor regrowth in bilateral neck masses and in the region of his left mandible	Subcentimeter pulmonary nodules and a soft tissue mass obstructing the right bronchus as well as a contralateral hilar metastasis	10 years	No	Wide surgical resection and jaw reconstruction/removal of a 7.5 × 5.5-cm tumor mass [27 years old]/resection of separate 8.0 and 7.0 cm recurrent tumor masses (31 years old)/field radiation therapy (33 years old)
None	Large, deforming mandibular mass preventing jaw closure and displacing the tongue to the left, resulting in severe dysphagia with inability to take nutrition by mouth, inability to control oral secretions, and poor speech intelligibility/swallowing showed overt aspiration of all consistencies	3.79 × 5.87 × 5.62 cm mass of the right mandibular body	67 years	No	Twice treated with conservative surgical approaches
Surrounding the residual tumor and in what used to be the core area of the original tumor, we found novel bone formation, both trabecular and cortical bone. Samples of bone taken at the original margin of the tumor, showed bone entirely free of tumor. BRAF‐mutated alleles were still noted, yet, with significantly decreased variant allele frequency	Not reported	Not reported	15 years	Yes	Not reported
Surrounding the residual tumor and in what used to be the core area of the original tumor, we found novel bone formation, both trabecular and cortical bone. Samples of bone taken at the original margin of the tumor, showed bone entirely free of tumor. BRAF‐mutated alleles were still noted, yet, with significantly decreased variant allele frequency	Not reported	Not reported	13 years	Yes	Not reported
Surrounding the residual tumor and in what used to be the core area of the original tumor, we found novel bone formation, both trabecular and cortical bone. Samples of bone taken at the original margin of the tumor, showed bone entirely free of tumor. BRAF‐mutated alleles were still noted, yet, with significantly decreased variant allele frequency	Not reported	Not reported	10 years	Yes	Not reported
None	Massive dyspnoea with a combined restrictive and obstructive syndrome	>30 bilaterlal lung metastases increasing in size	2 years	No	Wide surgical resection
None	Not reported	MRI revealed a lesion measuring 24 × 21 × 19 mm (2016)	7 years	No	Wide surgical resection in 1997/several surgical approaches that were consistently followed by disease recurrence/local radiotherapy with 24 years old

**Fig. 1:**
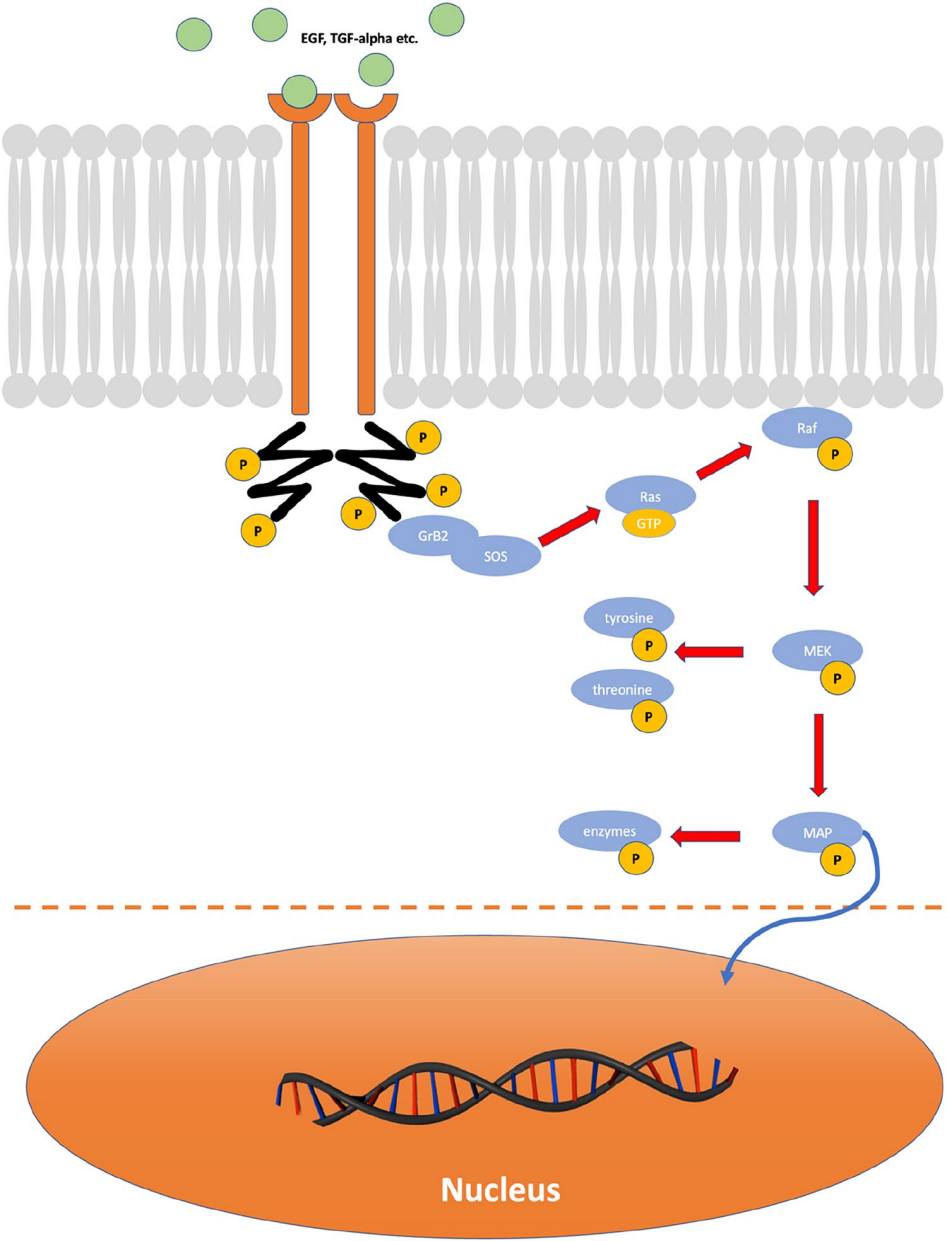
BRAF pathway: from the outer membrane to the nucleus

The patient population covers the entire expected spectrum in terms of both demographic- and disease-related characteristics. The youngest treated patient was 10 years old, and the oldest patient was 86 years old. There was an almost homogeneous distribution of women and men, with a distribution of 4:5. There were patients with an initial diagnosis of ameloblastoma, as well as patients with recurrences or metastasized ameloblastoma patients (initial diagnosis and recurrences). The indications here covered neoadjuvant therapy up to the use in multiple metastasized patients in an irresectable state. The results of off-label therapy corresponded to the broad spectrum of use. From “only” a tumor size reduction to restitutio ad integrum was reported (Fig. 1).

For example, Hirschhorn et al. reported three cases in children aged 10, 13, and 15 years with initial diagnosis of mandibular ameloblastoma. Here, neoadjuvant administration of Dabrafenib 4.5 mg/kg per day was given for durations of 15, 16, and 20 months, respectively. This reduced the size of the tumor to such an extent that complete resection of the tumor could subsequently take place without removal of the temporomandibular joint and without continuity resection of the mandible. The follow-up period of up to 38 months showed no evidence of tumor recurrence. Histologic workup revealed novel bone surrounding the residual tumor and the former core area of the original tumor. Both trabecular bone and cortical bone were evident. BRAF-mutated alleles still showed up in the molecular genetic analysis, but at a significantly reduced allele frequency [23].

A similar result was observed by Tan et al. The patient also received Dabrafenib monotherapy, but this was only for 73 days, as the patient discontinued therapy due to side effects. This was followed by composite resection of the tumor with titanium plate placement and pectoralis major skin flap. 90% of the tumor volume was reduced using the BRAF inhibitor and the BRAF-mutated alleles also appeared in significantly lower frequency. A special feature of this case is that histologically substantially greater areas of squamous differentiation were found in the treated specimen [24].

In particular, Brunet et al. demonstrated the efficacy of the combination therapy of Dabrafenib and Trametinib in recurrent metastatic ameloblastoma. The 26-year-old patient clinically presented with hemoptysis and cough 13 years after her initial ameloblastoma resection. With CT-diagnosed bipulmonary space-occupying lesions that later proved to be metastases from the ameloblastoma, she began combination therapy with Dabrafenib and Trametinib. Complete remission of the pulmonary metastases was seen after only 12 weeks. The therapy was ultimately continued for a total of 30 months [25].

Even a reduction of the dose with continued efficacy can be found in the literature. In the case of Faden et al., the 83-year-old woman had her dose of Dabrafenib reduced to only 75 mg due to her functional status and general disease. Despite this, monthly follow-up visits showed a significant decrease in tumor mass in the mandible. The initial dysphagia as well as the limited mouth closure due to the large mass also regressed significantly, allowing the patient to regain oral food intake [26].

Broudic-Guibert et al. also demonstrated good efficacy for Vemurafenib. It was used in a 33-year-old patient who had already undergone a mandibular resection at the age of 2 years due to an ameloblastoma. At the age of 33, he was presented with progressive dyspnea due to > 30 bilateral lung metastases. Here, too, the dose had to be halved due to arthralgias, nausea, and skin rash. Radiological follow-up showed a 30% reduction of tumor volume and a significant improvement of initial obstructive and restrictive lung problems [27].

Finally, Kaye et al. showed for the massively metastasized irresectable state the effect of the combination therapy Dabrafenib and Trametinib. The 40-year-old patient was already operated 3 times due to an ameloblastoma recurrence and underwent radiation once. Now he presented again due to a recurrence with bilateral neck masses, pulmonary nodules and a soft tissue mass obstructing the right bronchus as well as a contralateral hilar metastasis. Again, the combination therapy consisted of Dabrafenib and Trametinib. Already 8 weeks after the start of therapy, no FDG-18-enriching space-occupying lesions were detectable in the lung. Tumor masses in the neck and jaw were also significantly regressed. Even after 20 weeks of therapy, the tumor showed regression in all areas [28].

**Fig. 2:**
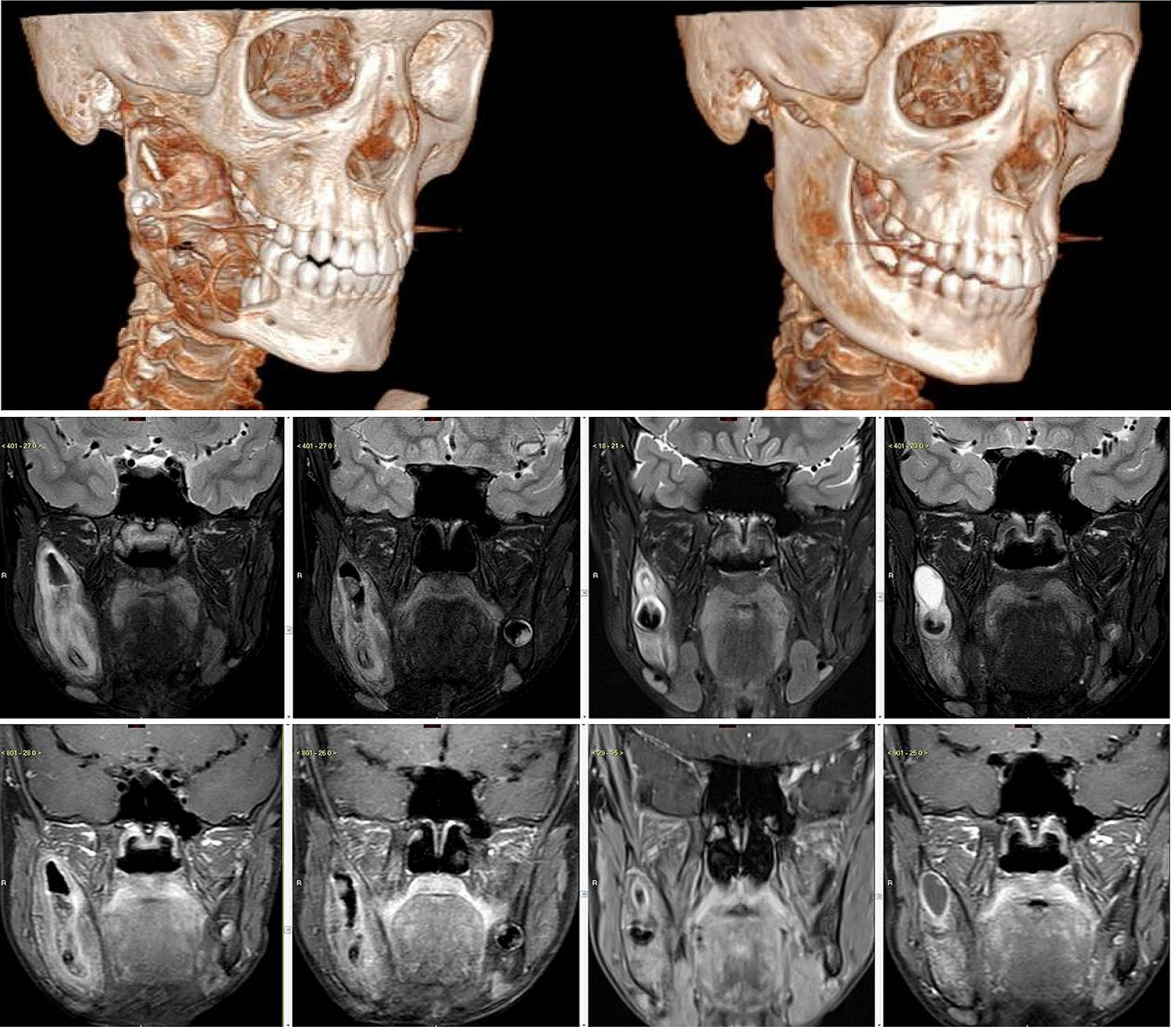
Top row shows the reconstructed 3-dimensional computerized tomography demonstrating on the left an osteolytic lesion involving the right mandibular body and ramus. On the right the partial radiologic response is demonstrated, observed after 10 months of targeted therapy which brought the mandible to a near pre-disease state. Middle and bottom rows—a consecutive series of MR images at different time points along the treatment course, from left to right, including Coronal T2 Fat saturated images (middle row) and corresponding Coronal T1 post-contrast Fat-suppressed images (bottom row). The Earliest scan from Oct 2018 (Image 1) shows the heterogeneous, mixed solid, and cystic nature of the intramedullary lesion, with resultant Bucco–lingual expansion of the body and ramus of the right mandible. One can notice the noticeable gradual reduction of the tumor mass with accompanying improvement of the bony contour, till the Nadir at June 2019 (Image 3). A 4th follow-up scan performed on Nov 2019 showed a suspected recurrent cystic lesion distal to the tooth bud, which upon curettage, eventually represented histologically proven pseudo-progression

**Fig. 3:**
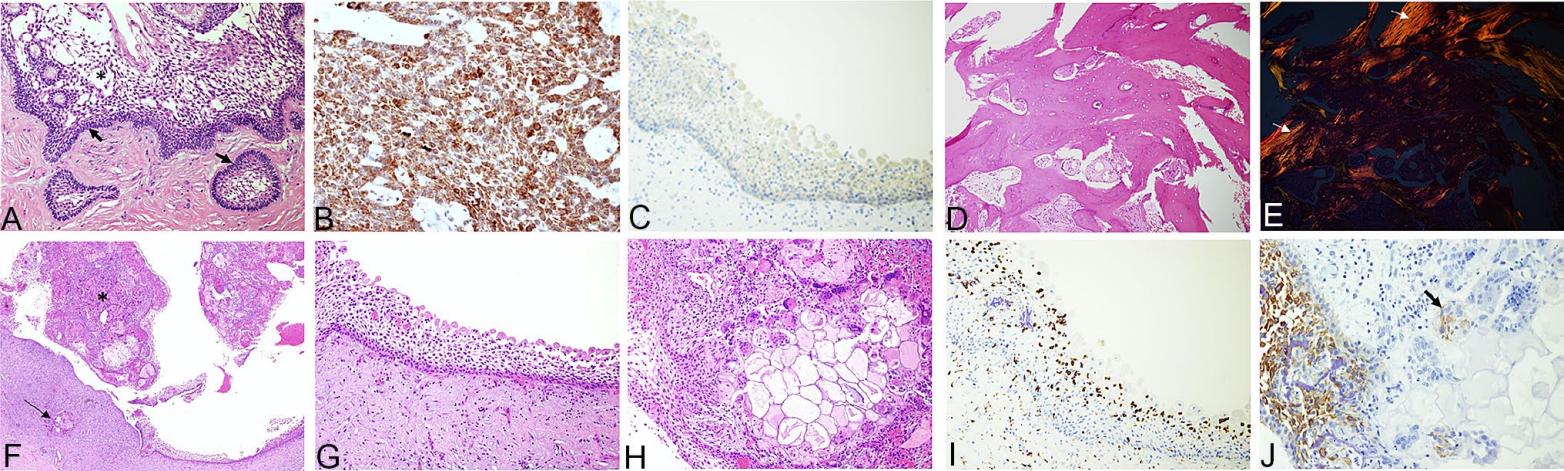
Top row: (**A**, **B**) Tumor at initial diagnosis. **A** Photomicrograph of one of the initial lesions of unicystic ameloblastoma—mural type, showing typical basal cells with a palisading pattern (arrows) and stellate reticulum-like appearance of the more superficial layers (asterisk) of the cystic epithelium (HE, original magnification X200). **B** BRAF immunostain of the initial lesion showing diffuse and strong reaction (BRAF, original magnification ×200). Tumor after anti-BRAF therapy. **C** BRAF immunostain shows only weak reaction of the ameloblastic lining epithelium (BRAF, original magnification ×200); Novel bone. **D** The bone at the periphery of the residual lesion was viable, of woven type and free of disease (HE, original magnification ×100). **E** The same field as in D seen with polarized light. The arrows point to small fragments of lamellar bone, while the main bone mass is of woven type (HE and polarized light, original magnification ×100); bottom row: Targeted therapy-mediated immune response (**F).** The persistent cystic lesion lined by ameloblastic epithelium. The lumen contains sheets of lining epithelium admixed with an inflammatory reaction predominated by multinucleated giant cells (asterisks) shown at a higher magnification in (**H**). In addition, the connective tissue wall contains many aggregates of multinucleated giant cells (arrow) (HE, original magnification ×40). **G** Major architectural alterations of tumor—The lining ameloblastic epithelium is devoid of the characteristic basal cell palisading pattern, and the other layers show dis-cohesion and/or changes indicative of apoptosis, which consist of an eosinophilic cytoplasm and small and pyknotic, almost absent, nucleus (HE, original magnification ×200). **H**–**J** Inflammatory reaction (**H**). The solid aggregate in the lumen from (**F**) at a higher magnification is composed of disintegrating ameloblastic epithelium admixed with numerous inflammatory cells, multinucleated giant cells, and hemorrhagic areas (HE, original magnification ×200); I CD68 (marker of macrophages) showing remarkable infiltration into the ameloblastic lining epithelium; numerous positively stained cells are also seen within the connective tissue wall (CD68, original magnification ×200). **J** CK19 immunostain (marker of odontogenic epithelium) highlights residual ameloblastic epithelium. One of the multinucleated giant cells contains remnants of CK19-stained fragments (arrow) (CK19, original magnification ×400)

Thus, we currently see a very low use of BRAF inhibitors in V600E-mutated ameloblastoma in the literature. However, the success of the therapy is clearly shown in all age groups and all tumor stages. For clinical and histological examples see Figs. 2 and 3.

## Discussion

Considering the fact that depending on the study, on average 2/3 of ameloblastoma show the BRAF-V600E mutation and that the standard therapy currently does not include a standardized drug therapy, and the data of the case reports cannot be given enough value. Therefore, BRAF inhibitor therapy should at least be considered in the treatment of ameloblastoma if the mutation is detected, especially in view of the fact that there is no standard therapy for metastatic ameloblastoma. Furthermore, the toxicity of such a mono- or combination therapy is generally good with the most common adverse events being rash, pyrexia, asthenia, headache, nausea, and arthralgia [29]. Even the use in children showed an acceptable side effect profile, so that the therapy with BRAF-inhibitors seems to be a conceivable therapy alternative to surgery. Especially, the complete response of some patients highlights the potential benefits of BRAF-targeted therapy. Yet, some molecular study showed that the residual disease still harbored BRAF mutation, although with a significantly decreased variant allele frequency. Collectively, this indicated that short-term BRAF monotherapy is highly effective but probably not curative [24]. This can be seen as an explanation for the frequent recurrences of ameloblastoma. However, it also underscores that BRAF inhibitors should perhaps not necessarily be used as the sole therapy at this time. Rather, their use to reduce tumor size with consecutive surgical treatment seems reasonable. This can significantly reduce the extent of surgery and save patients, especially children, from often mutilating surgery for far advanced findings. BRAF inhibitors simplify the already difficult and complex procedures to restore facial structures with respect to aesthetical aspects [23]. In addition, neoadjuvant use reduces the incidence of local recurrence in cases with limited surgical options. The only decisive factor seems to be a sufficient duration of therapy, as often a slow clinical response is observed due to the low proliferation index of ameloblastoma. Often amplified by alternative oncogenic pathways that evade BRAF inhibitor therapy. Here, fixed cut-off values need to be defined at which either therapy can be stopped or surgical resection of the finding should be performed [24].

## Conclusion

It should always be remembered that the face is essential to one’s perceived personal identity and is the primary organ of interpersonal communication and social interaction. Especially in children, ameloblastoma might result in impaired facial development, and disfigurement and profoundly impact of quality of life. Although long-term studies on BRAF inhibitors in BRAF-V600E-mutated ameloblastoma are lacking, we see their use to reduce tumor size with consecutive surgical treatment as a reasonable option for therapy. However, we are aware that at present the data are based only on case reports with the longest follow-up of just 38 months. The authors encourage the development of clinical trials in the use of BRAF Inhibitor drugs for select ameloblastoma patients in a high-volume multi-center setting.

## Data Availability

The datasets generated during and/or analysed during the current study are available from the corresponding author on reasonable request.

## References

[1] Effiom O, Ogundana O, Akinshipo A, Akintoye S (2018). Ameloblastoma: current etiopathological concepts and management. Oral Dis.

[2] McClary AC, West RB, McClary AC, Pollack JR, Fischbein NJ, Holsinger CF (2016). Ameloblastoma: a clinical review and trends in management. Eur Arch Otorhinolaryngol.

[3] Wenig BM. Atlas of head and neck pathology [Internet]. 2016 [cited 2022 Apr 10]. Available from: https://nls.ldls.org.uk/welcome.html?ark:/81055/vdc_100051463854.0x000001

[4] Becelli R, Carboni A, Cerulli G, Perugini M, Iannetti G. Mandibular ameloblastoma: analysis of surgical treatment carried out in 60 patients between 1977 and 1998. J Craniofac Surg. 2002;13(3):395–400. 10.1097/00001665-200205000-00006 12040207

[5] Carlson ER, Marx RE (2006). The ameloblastoma: primary, curative surgical management. J Oral Maxillofac Surg.

[6] Vayvada H, Mola F, Menderes A, Yilmaz M (2006). Surgical management of ameloblastoma in the mandible: segmental mandibulectomy and immediate reconstruction with free fibula or deep circumflex iliac artery flap (evaluation of the long-term esthetic and functional results). J Oral Maxillofac Surg.

[7] Zwahlen R (2002). Maxillary ameloblastomas: a review of the literature and of a 15-year database. J Cranio-Maxillofac Surg.

[8] Brown NA, Rolland D, McHugh JB, Weigelin HC, Zhao L, Lim MS (2014). Activating *FGFR2–RAS–BRAF* Mutations in Ameloblastoma. Clin Cancer Res.

[9] Kurppa KJ, Catón J, Morgan PR, Ristimäki A, Ruhin B, Kellokoski J (2014). High frequency of BRAF V600E mutations in ameloblastoma. J Pathol.

[10] Sweeney RT, McClary AC, Myers BR, Biscocho J, Neahring L, Kwei KA (2014). Identification of recurrent SMO and BRAF mutations in ameloblastomas. Nat Genet.

[11] Diniz MG, Gomes CC, Guimarães BVA, Castro WH, Lacerda JCT, Cardoso SV (2015). Assessment of BRAFV600E and SMOF412E mutations in epithelial odontogenic tumours. Tumor Biol.

[12] Wright JM, Soluk Tekkeşin M. Odontogenic tumors. Where are we in 2017? J Istanb Univ Fac Dent. 2017. 10.17096/jiufd.52886 PMC5750825 29354306

[13] Maurer G, Tarkowski B, Baccarini M (2011). Raf kinases in cancer–roles and therapeutic opportunities. Oncogene.

[14] Peyssonnaux C, Eychène A (2001). The Raf/MEK/ERK pathway: new concepts of activation. Biol Cell.

[15] Cuevas BD, Abell AN, Johnson GL (2007). Role of mitogen-activated protein kinase kinase kinases in signal integration. Oncogene.

[16] McCubrey JA, Steelman LS, Chappell WH, Abrams SL, Wong EWT, Chang F (2007). Roles of the Raf/MEK/ERK pathway in cell growth, malignant transformation and drug resistance. Biochim Biophys Acta BBA Mol Cell Res.

[17] Steelman LS, Pohnert SC, Shelton JG, Franklin RA, Bertrand FE, McCubrey JA (2004). JAK/STAT, Raf/MEK/ERK, PI3K/Akt and BCR-ABL in cell cycle progression and leukemogenesis. Leukemia.

[18] Morrison DK (2012). MAP kinase pathways. Cold Spring Harb Perspect Biol.

[19] Girotti MR, Lopes F, Preece N, Niculescu-Duvaz D, Zambon A, Davies L (2015). Paradox-breaking RAF inhibitors that also target SRC are effective in drug-resistant BRAF mutant melanoma. Cancer Cell.

[20] Cohen JV, Sullivan RJ (2019). Developments in the space of new MAPK pathway inhibitors for BRAF-mutant melanoma. Clin Cancer Res.

[21] European Medicines Agency. Tafinalar: EPAR—Product information. 2021.

[22] European Medicines Agency. Zelboraf: EPAR—Product information. 2021.

[23] Hirschhorn A, Campino GA, Vered M, Greenberg G, Yacobi R, Yahalom R (2021). Upfront rational therapy in BRAF V600E mutated pediatric ameloblastoma promotes ad integrum mandibular regeneration. J Tissue Eng Regen Med.

[24] Tan S, Pollack JR, Kaplan MJ, Colevas AD, West RB (2016). BRAF inhibitor treatment of primary BRAF -mutant ameloblastoma with pathologic assessment of response. Oral Surg Oral Med Oral Pathol Oral Radiol.

[25] Brunet M, Khalifa E, Italiano A (2019). Enabling precision medicine for rare head and neck tumors: the example of BRAF/MEK targeting in patients with metastatic ameloblastoma. Front Oncol.

[26] Faden DL, Algazi A (2017). Durable treatment of ameloblastoma with single agent BRAFi Re: Clinical and radiographic response with combined BRAF-targeted therapy in stage 4 ameloblastoma. JNCI J Natl Cancer Inst..

[27] Broudic-Guibert M, Blay JY, Vazquez L, Evrard A, Karanian M, Taïeb S (2019). Persistent response to vemurafenib in metastatic ameloblastoma with BRAF mutation: a case report. J Med Case Reports.

[28] Kaye FJ, Ivey AM, Drane WE, Mendenhall WM, Allan RW. Clinical and radiographic response with combined BRAF-targeted therapy in stage 4 ameloblastoma. JNCI J Natl Cancer Inst. 2014;107(1):dju378. 10.1093/jnci/dju378 25475564

[29] Knispel S, Zimmer L, Kanaki T, Ugurel S, Schadendorf D, Livingstone E (2018). The safety and efficacy of dabrafenib and trametinib for the treatment of melanoma. Expert Opin Drug Saf.

